# Bleeding Complications in Anticoagulated and/or Antiplatelet-Treated Patients at the Dental Office: A Retrospective Study

**DOI:** 10.3390/ijerph18041609

**Published:** 2021-02-08

**Authors:** Esther Martínez-Moreno, Federico Martínez-López, Francisco Javier Rodríguez-Lozano, Ricardo Elías Oñate-Sánchez

**Affiliations:** Special Care in Dentistry and Gerodontology Unit, Department of Dermatology, Stomatology, Radiology and Physical Medicine, Morales Meseguer Hospital, Faculty of Medicine, University of Murcia, 30100 Murcia, Spain; esthermartinezmoreno@gmail.com (E.M.-M.); femarlodent@gmail.com (F.M.-L.); reosan@um.es (R.E.O.-S.)

**Keywords:** anticoagulated, antiplatelet-treated, simple extractions, bleeding complications

## Abstract

Background: Bleeding complications in patients undergoing antiplatelet and/or anticoagulant therapy have been one of the main concerns in dental practice. Upon the introduction of new antiplatelet and anticoagulant drugs, there is a search for new protocols that respond to a secure treatment. The aim of the present study was to evaluate bleeding complications in anticoagulated and antiplatelet-treated patients after performing simple dental extractions, in a period of 4 years. Material and Methods: 147 clinical records of anticoagulated and/or antiplatelet-treated patients undergoing a simple dental extraction over a period of 4 years (October 2015 to September 2019) were studied. Within the sample, 63 patients were antiplatelet-treated, 83 were anticoagulated, and 1 patient was under both therapies. Within the anticoagulated patients, 70 took classic anticoagulants and 14 new oral anticoagulants (NOACs). Quantitative data were studied with arithmetic mean and standard deviation (SD). The chi-square test was used for the qualitative variables. ANOVA tests were used to compare age and anticoagulated or antiplatelet-treated patients. Statistical significance was determined when *p* < 0.05. Results: From the 418 dental extractions performed, five severe bleeding complications took place in three patients (2.11%). From the five events, four were in patients treated with NOACs (1.68%) and one occurred in a patient anticoagulated with acenocoumarol (0.42%; *p* = 0.003). Conclusions: Considering the results of this retrospective clinical study, we can conclude that bleeding complications in anticoagulated and/or antiplatelet-treated patients after tooth extractions were low, with a higher incidence recorded in patients treated with NOACs, followed by classic anticoagulants, and there were no complications in antiplatelet-treated patients.

## 1. Introduction

Anticoagulants and antiplatelet agents are widely used in patients with high thromboembolic risk [1]. These drugs are commonly administered in patients who present cardiovascular diseases [1], arrhythmias such as atrial fibrillation [1,2], neurological disorders including embolic accident or transient ischemic attack (TIA) [1,2], as well as surgeries where the immobilization period is expected to be long [1]. They are also used in cases of pulmonary embolism and deep vein thrombosis [2]. In this way, thromboembolic events are controlled and prevented in high-risk patients [1]. When treating patients under anticoagulant and/or antiplatelet agents at the dental office, the drugs administered to the patient must be analyzed. Additionally, before performing dental procedures where bleeding is to be expected, the individualized risk of bleeding of the patient as well as the cause for the administration of these agents need to be identified. As Cervino et al. [3] described, studying the patient’s main systemic disease leads us to an accurate planification of the dental treatment according to the medical condition, thereby minimizing risks.

One of the classic anticoagulant agents is heparin, which is administered parenterally. Low-molecular-weight heparins (LMWH) are usually applied as bridging therapy in anticoagulated patients when a surgery or long immobilization is planned, and when anticoagulation is required for a limited period of time. This drug presents an antidote: protamine sulphate [1].

Alternatively, we find anticoagulant agents such as acenocoumarol and warfarin. Both are coumarin anticoagulants, also known as Vitamin K antagonists (VKAs) [1,4]. Their action can be modified by factors such as the duration of the treatment or even diet. They present numerous drug interactions and are quantified by an International Normalized Ratio (INR) [1,5].

New oral anticoagulants (NOACs) were introduced with the goal of reducing the interactions from the classic anticoagulants. They present a more direct and selective way of action. However, unlike the old anticoagulants, they do not have a specific monitoring test to evaluate their effect [4,5,6]. We find anticoagulants such as dabigatran, with renal elimination, contraindicated in severe renal impairment [1,4]. It presents a half-life of 12 to 17 h [5,7] and its peak concentration in plasma is reached 2–3 h after its administration [5]. It also presents an antidote: idarucizumab [4,8]. On the other hand, rivaroxaban presents a plasma half-life of 7 to 13 h [5], reaching its maximum peak after 2–4 h [5,7]. Its antidote is andexanet alfa [8]. Apixaban presents a half-life of 8 to 13 h, with its peak after 3–4 h after administration [7]. Andexanet alfa is also the antidote in charge of the reversion of this anticoagulant’s action [8]. Lastly, we find edoxaban, found in plasma from 10 to 14 h [5,7] and reaching its plasmatic peak at 1.5 h [7].

Within the classic antiplatelet agents, we find acetylsalicylic acid (ASA), which exhibits an anti-inflammatory and antiplatelet activity. It is one of the most used drugs for the prevention of thromboembolic events. Its action corresponds to the platelet’s half-life: from 7 to 10 days. On the other hand, we find clopidogrel, which presents an irreversible action from 7 to 10 days. Its main use is in patients with acute myocardial infarction (AMI) [1], as well as with unstable angina [3], normally administered alone or associated to ASA. The classic antiplatelet drugs have presented some disadvantages: high risk of bleeding, the presence of residual risk of ischemic diseases, or the variability of the drug’s antiplatelet effect. Thus, new antiplatelet agents which do not increase the risk of bleeding at the expense of reducing the patient’s thromboembolic risk were searched for. Among them, we find ticagrelor. It achieves an antiplatelet action after 2 h of its administration and its effect disappears 12 h later [1].

Due to the appearance of new antiplatelet agents/anticoagulants, the knowledge of the potential hemorrhagic and/or thrombotic effects that may occur at the dental office is a matter of interest [1,2,3,4,5,6,7,8]. Accordingly, the aim of the present retrospective study was to evaluate bleeding complications in anticoagulated and antiplatelet-treated patients during simple dental extractions in a period of 4 years.

The specific objectives were to unify the clinical management of anticoagulated and/or antiplatelet-treated patients when performing simple dental extractions, to identify risk groups among the sample, to describe which anticoagulant and/or antiplatelet drugs were mostly used in the sample, to value the final clinical acts, to study which teeth were the most extracted in the study sample, to study those cases that presented bleeding complications after tooth extraction, to value the usefulness of local hemostatic measures in these patients, and to plan the dental act according to the anticoagulant/antiplatelet agent’s pharmacodynamic characteristics.

## 2. Material and Methods.

Clinical records of anticoagulated and antiplatelet-treated patients undergoing simple dental extractions from October 2015 to September 2019 were evaluated. Age, gender, simple or multiple antiplatelet treatment and/or anticoagulant treatment, number of dental extractions, the teeth that were extracted, number of total visits, and bleeding complications of each patient were recorded. The inclusion criteria for the patients studied were as follows: patients under anticoagulant therapy, patients under antiplatelet treatment, or patients under both therapies, who required at least one simple dental extraction and were in good medical condition for the performance of the surgery. The exclusion criteria included patients who were not taking anticoagulants or antiplatelet drugs, patients who did not require a simple dental tooth extraction, and medically uncontrolled patients.

Simple dental extractions imply from 1 to 3 dental pieces extracted, with minimal wound size [7] and no need of elevating any flap or damaging the alveolar bone [9]. The same protocol was followed for every patient and is reflected in Table 1.

In antiplatelet-treated patients, under single or dual therapy, pharmacological treatment was not suspended. In anticoagulated patients taking classic anticoagulants (acenocoumarol, warfarin, or heparin), the drug was not suspended and INR was monitored up to 72 h before the dental extraction [1]. In anticoagulated patients under treatment with NOACs, the specialist’s guideline (cardiologist or neurologist) was followed: it usually consisted on the suspension of the dose 24 h before the dental extraction and the reintroduction of the drug 6 h after the dental extraction, once hemostasis was achieved [10]. When planning the antibiotic therapy, antibiotic coverage was administered in those patients where it was indicated, after studying their systemic characteristics, as well as the local factors implicated. In this way, the appearance of adverse drug reactions and development of bacterial resistance were avoided [11,12].

The evaluation of bleeding complications was done following the criteria presented by Iwabuchi et al. [9]. After the tooth extraction, the patient was instructed to bite a gauze for 30 min, after which the bleeding was evaluated. Bleeding criteria are summarized in Table 2 [9].

The authors consider that the presence of bleeding complications is reached when a patient presents a level 2.2. or 3 [9].

A favorable report was obtained by the Clinical Research Ethics Committee to perform the present study (ID: 2815/2020). An Excel database (Microsoft Office for Windows 10, Washington, DC, USA) was elaborated for analysis. The statistical analysis was performed using IBM SPSS Statistics version 27.0 (IBM, Armonk, NY, USA). A descriptive study of the quantitative data was performed using arithmetic mean and standard deviation (SD). For the qualitative data, a chi-square test was performed. ANOVA was used twice, to compare age and anticoagulated patients, and age and antiplatelet-treated patients. The level of statistical significance adopted was *p* < 0.05.

## 3. Results

The sample was composed of 147 subjects: 86 men and 61 women (46 to 93 years old). The mean age of the sample was 74.03 years old. The group from 81 to 90 years old was the largest. According to gender, women’s mean age was 61 years old and men’s mean age was 75.95 (76 years old). Related to treatment, 63 patients were taking antiplatelet treatment, 83 were undergoing anticoagulant treatment, and 1 patient was under both therapies (acenocoumarol and ASA). The sample according to gender and pharmacological treatment is presented in Table 3. The descriptive study of the patients according to age is reflected in Table 4.

**Figure 1:**
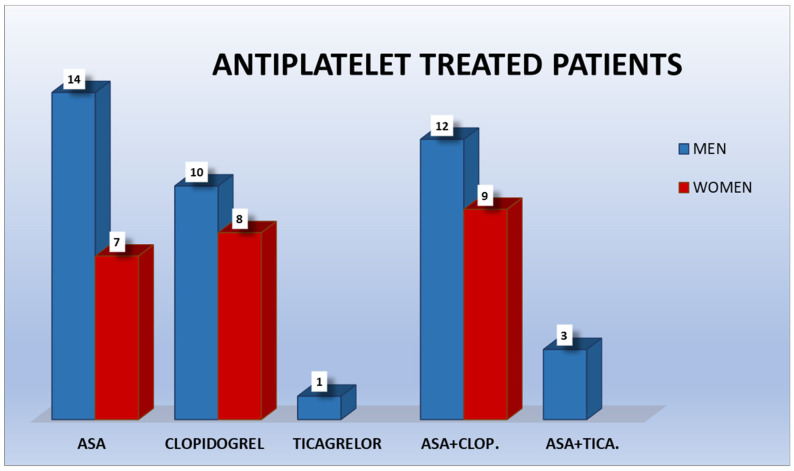
Antiplatelet-treated patients.

**Figure 2:**
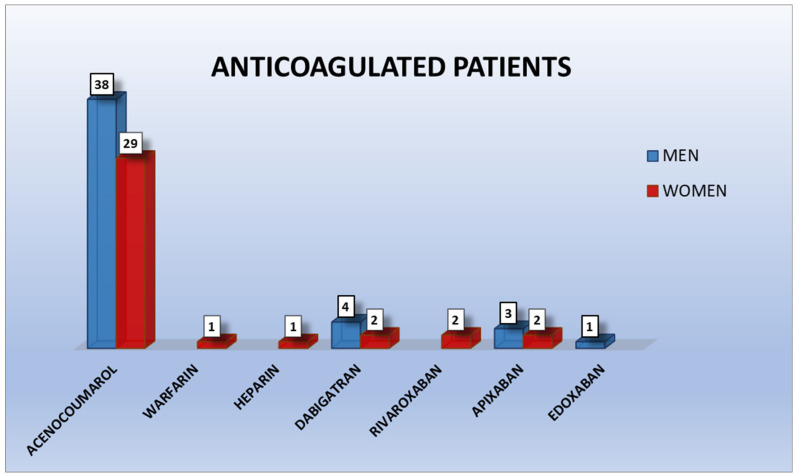
Anticoagulated patients.

Of the antiplatelet-treated patients, 21 took ASA, 18 clopidogrel, 1 ticagrelor, 21 ASA + clopidogrel, and 3 ASA + ticagrelor; therefore, 24 were under dual therapy. The group was also divided depending on gender (Figure 1). Of the anticoagulated patients, 70 took classic anticoagulants (VKAs and heparin) and 14 new oral direct anticoagulants (6 dabigatran, 2 rivaroxaban, 5 apixaban, and 1 edoxaban). The division of the sample following gender was also studied (Figure 2).

**Figure 3:**
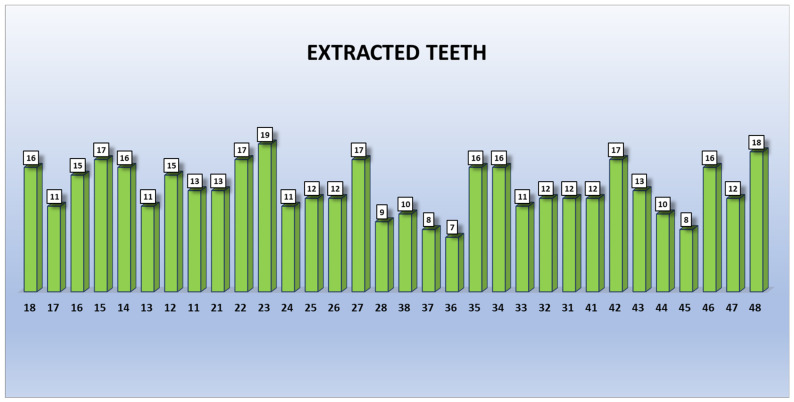
Number of extractions per tooth. Each tooth is represented by its Fédération Dentaire Internationale (FDI) numeric nomenclature.

A total of 418 dental extractions were performed in 318 visits. The most extracted tooth was—using Fédération Dentaire Internationale (FDI) nomenclature—2.3, followed by 4.8 (Figure 3). From the 418 tooth extractions, 180 were performed in 64 antiplatelet-treated patients (124 men and 56 women), 237 were carried out in 84 anticoagulated patients (127 men and 110 women), and 1 in a male anticoagulated and antiplatelet-treated patient. From the 237 dental extractions performed in anticoagulated patients, 181 were patients under classic anticoagulants and 56 in patients under NOACs. The INR in patients taking classic anticoagulants ranged from 1.3 to 3.6.

Severe bleeding complications were recorded in five tooth extractions (2.11% from the total extractions), which took place in three anticoagulated patients, according to grade 2.2 or 3 from Iwabuchi et al. [9] scale. From the five events, four were in patients treated with NOACs (1.68%) and one occurred in a patient anticoagulated with acenocoumarol (0.42%). The chi-square test for bleeding complications in patients undergoing NOACs resulted in a level of statistical significance of *p* = 0.003 (Table 5). This outcome must be interpreted carefully, due to the relative nature of the sample. In antiplatelet-treated patients, significant bleeding complications were not recorded.

## 4. Discussion

In the present retrospective clinical study of 147 patients, a total of 86 men (39 antiplatelet-treated, 46 anticoagulated, and 1 under both therapies) and 61 women (24 antiplatelet-treated and 37 anticoagulated) were presented. This study sample is comparable to that of other studies in the field. For example, in a study by Giudice’s et al. [13], a sample of 40 patients (28 men and 12 women) were examined. Every patient was antiplatelet-treated. Berton et al. [14] presented two groups: one comprising patients under VKA anticoagulant therapy with 31 men and 34 women, and another group of 34 men and 31 women treated with NOACs. Lastly, Tang et al. [15] reported a sample of antiplatelet-treated patients (182 men and 156 women), and Lu et al. [16] presented a study in 110 men and 73 women under antiplatelet treatment, and two groups of anticoagulated patients (15 women and 17 men under anticoagulated treatment who suspended warfarin therapy upon dental extraction, and another group with 12 women and 16 men who continued the therapy with warfarin). In general terms, the samples from similar studies are majorly represented by men as opposed to women, as seen in the present study.

Publications related to the dental management of patients undergoing NOAC therapy are limited. Among them, the study by Berton et al. [14] compared performing simple dental tooth extractions in patients under classic anticoagulants and those taking NOACs, without suspending the treatment with any of the drugs. They reported that the results were similar in both groups and reinforce what the most recent literature suggests, which is not to suspend the anticoagulant therapy with NOACs when a simple surgical treatment is planned. Nevertheless, this method is not standardized, and no protocol has been developed, as opposed to the available protocol for classic anticoagulants. Another study that compared managing patients under dabigatran, rivaroxaban and apixaban or warfarin is the one performed in 205 patients by Cabbar et al. [17]. However, they used an alternative method: they interrupted the pharmacological therapy or gave a bridging therapy upon dental extraction. Both Berton et al. and Cabbar et al. discuss the need for more studies in the field in order to elaborate a standardized protocol for the clinical management of these patients. Case reports are also available among the literature in the field. Ehrhard et al. [18], for example, presents a case report from a patient taking apixaban where five dental extractions were performed in one clinical act, having previously administering a bridging therapy to the patient. The patient presented profuse bleeding after the extraction. However, the patient presented an impaired kidney function, which affects the anticoagulant’s action, extending the anticoagulant effect in time. This highlights the importance of a critical assessment of the cases presented in the field. All of the underlying factors that may affect the patient’s hemostatic potential, other than the antiplatelet/anticoagulant therapy and its modification, need to be taken into consideration when interpreting the results.

Studies with a greater sample, such as Tang et al.’s [15], with a sample of 338 patients, who only took antiplatelet treatment (ASA, clopidogrel, or both), are also found among the literature, although they are limited. A case study proposal has been planned by Ockerman et al. [19], planning to evaluate the efficacy of using tranexamic acid in the postoperative bleeding of patients taking NOACs, in a sample of 236 patients.

When studying anticoagulated patients, some research projects compare patients taking NOACs against patients taking classic anticoagulants. Berton et al.’s [14] study, which has been mentioned previously, analyzed a final sample of 65 patients under classic anticoagulants and 65 patients taking NOACs. Brennan et al.’s [20] study is another example. Their sample was formed by 21 patients who took warfarin and 86 who took NOACs. Another study, which only includes NOACs, is the one lead by Cocero et al. [21]. They evaluated 100 patients between November 2015 and November 2017. Finally, studies like the present one are also found, in which postoperative bleeding in anticoagulated patients and antiplatelet-treated patients is compared. Lu et al. [16] studied a sample of 1331 patients, 60 anticoagulated by warfarin, from which 28 patients continued warfarin treatment and 32 stopped the treatment, taking heparin instead before the dental extraction. From the sample, 183 patients were under antiplatelet treatment: 42 were taking clopidogrel, 125 ASA, and 16 dual antiplatelet treatment. The rest of patients correspond to control patients who did not take any anticoagulant or antiplatelet agents: 1088 patients. So, the final sample of anticoagulated and/or antiplatelet-treated patients consisted of 243 patients. Nevertheless, they did not evaluate patients taking NOACs.

In the sample from the present study, 418 tooth extractions were performed in 318 clinical acts. Iwabuchi et al. [9] carried out a tooth extraction per patient. Their anticoagulated group was formed by 496 patients, and their control group, which did not take anticoagulant therapy, was formed by 2321 patients. A total of 2817 teeth were extracted. In the study by Lu et al. [16], they valuated 243 patients under antiplatelet or anticoagulant therapy. In their anticoagulated sample, in 32 patients who changed to bridging therapy, 130 teeth were extracted in 37 clinical acts. In the group of 28 patients who continued their warfarin therapy, 77 teeth were extracted in 33 dental appointments. In antiplatelet-treated patients, 548 teeth were extracted in a total of 274 clinical acts. Tang et al. [15] extracted 469 teeth from 338 patients who were under antiplatelet treatment. From the tooth extractions performed, 307 were simple extractions and 31 were complex. Brennan et al. [20] extracted teeth in anticoagulated patients, who were divided in two groups: one group comprising 21 patients taking warfarin where 50 dental pieces were extracted and another group with 86 patients taking NOACs where 145 teeth were extracted. Cocero et al. [21] performed extractions of 66 teeth in a group that took NOACs and did not presented associated comorbidities and extracted 72 teeth in the group that took NOACs and had associated comorbidities. Their dental extractions were divided into simple, dual, and triple adjacent teeth. They also describe if multirooted teeth were involved or not.

The criteria used in this study when evaluating bleeding complications correspond to that from the study by Iwabuchi et al. [9]. There are some studies that use this exact bleeding complication’s criteria [14,22]. Yagyuu et al. [23] state that their sample’s statistical analysis was based on Iwabuchi’s work [9]. Other studies follow their own bleeding criteria, like the study of Tang et al. [15]. They describe the event of late postoperative bleeding if the bleeding continued after the patient was kept 30 min biting the gauze. They also classify the bleeding as mild or severe, if the postextraction bleeding lasted less or more than 12 h, respectively. Brennan et al. [20] categorize bleeding as two types: major and minor bleeding. Major bleeding is distinguished when, after the tooth extraction, medical care or hospitalization was needed. The rest of the events would classify as minor bleeding events. Additionally, they weighed the gauze before and after the dental extraction, to record the patient’s immediate blood loss. Intraoperative blood loss was also recorded by weighing gauzes in the study lead by Gupta et al. [24]. Most of the articles considered an adequate hemostasis if bleeding is controlled after 30 min of biting a gauze [9,15,20,25]. Other authors, such as Mauprivez et al. [26], consider a bleeding complication when bleeding cannot be stopped after biting the gauze for 20 min, when having already used local hemostatic methods. Lu et al. [16] considered an immediate bleeding if the bleeding was not controlled after biting the gauze for 10 min.

Bleeding complications took place in five dental extractions in three patients from our sample: one tooth extraction in a patient taking acenocoumarol and four in patients under apixaban. Other authors highlight that from five cases of considerable bleeding in patients taking NOACs, three of them occurred in patients under rivaroxaban treatment [14]. They suggest higher bleeding risk with rivaroxaban, based also on evidence found in the literature [14]. Kwak et al. [27] performed different dental treatments, where bleeding was implied, in 120 patients: 53 women and 67 men. All of them were taking NOACs. Nine cases presented late bleeding, five cases with rivaroxaban: two of them in dental scaling, two of them in the first step of a dental implant placement, and one in a simple dental extraction. There was one case of dabigatran in the primary step of a dental implant placement, two cases of apixaban, both in simple tooth extractions, and one case of edoxaban in a resin filling restoration, a procedure that is considered to be of low risk of bleeding.

Lababidi et al. [28] compared two groups of patients: 59 under warfarin treatment and 53 patients taking NOACs. They performed different dental treatments that implied bleeding: simple and multiple dental extractions, surgical extractions, and biopsies. Within the group of patients taking NOACs, four cases presented bleeding complications. All of them belonged to a subgroup where the anticoagulation therapy was not modified. Biopsies were also performed in the study of de Almeida Barros Mourão et al. [29], who valued the efficacy of platelet-rich fibrin for achieving hemostasis after oral biopsies. Doganay et al. [30] obtained 11 patients with bleeding complications, from 222 evaluated: three presented moderated bleeding and eight a mild bleeding. From the antiplatelet-treated sample, bleeding frequency was higher in those patients under dual antiplatelet treatment. Nevertheless, they state that this difference has no statistic value, when compared to those patients under single antiplatelet therapy. Complementarily, Hamzah et al. [31] studied 60 patients, divided into three groups of 20: two groups where postoperative bleeding was evaluated in patients under antiplatelet treatment and/or anticoagulation who presented ventricular assist devices, and a third group who did not present these devices. They did not discontinue the drug therapy in any group. Two patients from the control group (*n* = 20) presented bleeding complications, compared to three patients from the experimental group (*n* = 40). In Yamada et al.’s [32] project, third molar extractions were performed in anticoagulated patients under warfarin. From 142 patients, 31 presented delayed bleeding: 17 events were categorized as a grade 2 (need for an additional suture of the wound or the use of a hemostatic agent) and 14 events were defined as a grade 1 (hemostasis was accomplished under compression). Saninno et al. [33] studied postoperative bleeding complications in patients undergoing “all on four” rehabilitations. The placement of four implants took place in three different groups: patients taking warfarin, patients taking rivaroxaban, or those with no therapy. A total of 11 moderate bleeding complications were recorded in the group taking warfarin, three complications in patients taking rivaroxaban, and 1 complication in the control group. The study also recorded numerous mild bleeding complications and valued if postoperative purpura was present after surgery.

In another retrospective study of patients who underwent a dental extraction by another practitioner, a total of 542 patients who attended an emergency unit due to presenting delayed bleeding were assessed. Moderate bleeding events were more frequent in patients taking LMWH. From the 19 cases of severe bleeding, they were mostly associated to patients who took warfarin or LMWH. In 42 cases, hospitalization was needed, and the remaining 500 patients abandoned the unit after the bleeding was controlled 1 h later [34]. In the present study, a percentage of 2.11% of bleeding complications was obtained from the sample. Febbo et al. [35] recorded a similar result: nine patients presented bleeding complications in their sample of 439 anticoagulated patients, which represents 2.1%. Iwabuchi et al. [9] obtained 3.6% bleeding complications, which corresponded to grades 2.2 or 3 following their criteria, from a total sample of 496 anticoagulated patients.

As stated previously, the present study sample was composed of 70 patients who took classic anticoagulants and 14 who were treated with new oral direct anticoagulants. This could be considered as a limitation when having to compare both groups, since the difference in the number of patients is substantial. Nevertheless, this difference has also been seen in similar studies, as described previously. In addition, the distribution of the sample corresponds to the general distribution of patients undergoing anticoagulated treatments in our population area, thereby increasing the validity of the study.

The authors declare that there are no conflicts of interest.

## 5. Conclusions

Considering the results of this retrospective clinical study, we can conclude that bleeding complications in anticoagulated and/or antiplatelet-treated patients after tooth extractions were low, recording a higher incidence in patients treated with NOACs, followed by those treated with classic anticoagulants, and no complications in antiplatelet-treated patients. More studies are needed with larger samples and standardized methodologies, so new scientific evidence about the clinical management of these new drugs can be generated.

## Figures and Tables

**Table 1:** Extraction protocol.

Treatment of infection or inflammation using antibiotic coverage and/or chlorhexidine mouthwash before the tooth extraction [1].
Atraumatic surgery, bone regularization, and removal of the granulation tissue [1].
Dental suturing [1].
Alveolus irrigation using tranexamic acid [1].
Biting an tranexamic acid-dampened gauze [1] for 30 min [9].
Monitoring International Normalized Ratio (INR) a maximum of 72 h prior to dental extraction in Vitamin K antagonists (VKAs) anticoagulated patients [1].

**Table 2:** Bleeding criteria.

Grade	Clinical Correlation
0	No bleeding.
1	Adequate hemostasis, blood clot is present.
2.1	Hemostasis was reached after the compression of the wound for more than 30 min.
2.2	Blood oozing in regions where hemostasis was achieved the next day or the same day of the extraction using compression.
3	Hemostasis was achieved by procedures that differ from compression.

**Table 3:** Sample characteristics.

Gender	Antiplatelet Therapy	Anticoagulant Therapy	Both	Total
Women	24	37	0	61
Men	39	46	1	86
Total	63	83	1	147

**Table 4:** Arithmetic mean ($\bar{x}$) and standard deviation (SD) according to age (years).

	Women (*n* = 61)		Men (*n* = 86)		Total (*n* = 147)	
Age	$\bar{x}$	SD	$\bar{x}$	SD	$\bar{x}$	SD
41–50	0	0	46	0	46	0
51–60	53.400	1.673	56.444	3.395	55.357	3.201
61–70	65.846	3.648	65.115	2.930	65.359	3.158
71–80	74.600	2.720	74.667	2.443	74.641	2.518
81–90	84.375	2.337	84.500	2.844	84.437	2.576
91–100	91.500	1	91.500	0.707	91.500	0.837

**Table 5:** Chi-square tests for bleeding complications in patients taking new oral anticoagulants (NOACs).

Test	Value	Degrees of Freedom	Bilateral Asymtotic Significance
Pearson’s chi-square	11.944	2	0.003
Likelihood ratio	7.323	2	0.026
Linear-by-linear association	2.276	1	0.131

## Data Availability

Not applicable.
